# Quantification of an Elite Futsal Team’s Microcycle External Load by Using the Repetition of High and Very High Demanding Scenarios

**DOI:** 10.3389/fpsyg.2020.577624

**Published:** 2020-10-15

**Authors:** Jordi Illa, Daniel Fernandez, Xavier Reche, Gerard Carmona, Joan Ramon Tarragó

**Affiliations:** ^1^Sports Performance Area, Sport Science Department, Futbol Club Barcelona, Barcelona, Spain; ^2^Escola Superior de Ciències de la Salut Tecnocampus, Universidad Pompeu Fabra, Mataró, Spain

**Keywords:** most demanding scenario, team sport, game analysis, training microcycle, ultra-wideband

## Abstract

The main objective of this study was to describe the repetition of external load high-demanding scenarios and very high-demanding scenarios of match play for velocity, distance, and neuromuscular locomotor variables of an elite futsal team. Additionally, we also checked how these high- and very high-demanding scenarios were distributed throughout the microcycle. The most demanding scenario (measured using a rolling average method with a 1-min time window) of match play was measured out of thirteen elite futsal players using a local positioning system in the course of thirteen official matches and six in-season microcycles. A mean of the top three match play observations for each variable and each player were used to determine the most demanding scenario (100%) reference value. Data were reanalyzed to count the number of high-demanding scenarios (80–90% of the individual most demanding scenario) and very high-demanding scenarios (>90% of the individual most demanding scenario). The number of scenarios was analyzed with respect to the number of days prior to the match [match day (MD) minus X] and a bootstrap confidence interval approach was used to assess differences between MD. During a single match, players have to cope with repeated high- and very high-demanding scenarios. Moreover, the training session 2 days prior to the match was the one most similar to the match, surpassing it only in scenarios of locomotor velocity variables, albeit with significantly fewer scenarios of neuromuscular variables. The number of high- and very high-demanding scenarios in the training session prior to the match dropped significantly in comparison with the rest of the microcycle and the match. This new monitoring method may help practitioners to establish an accurate assessment of external load demands in competition and training.

## Introduction

With a view to objectively preparing players for what they may encounter during competition, coaches and strength and conditioning coaches have broadly based training prescription on match activity profile, commonly derived from the average demands of match play. However, this approach is likely to result in players being underprepared for the most demanding scenario of match play (Gabbett et al., 2016). This seems to be a common concern for most practitioners who, regardless of the method, have sought to develop alternative approaches to identify the most demanding scenario of match play, since such consideration seems to contribute to the implementation of a more accurate training program prescription (Gabbett et al., 2012). Rolling average methods have commonly been used to describe the peak periods of competition in different sports such as Australian football (Delaney et al., 2017), soccer (Malone et al., 2015; Martín-García et al., 2020), basketball (Vázquez-Guerrero et al., 2020), and rink hockey (Fernández et al., 2020).

The interest among practitioners in quantifying high-intensity running and effort bouts is not new. Some well-known examples are concepts such as repeated-sprint ability and repeated-effort ability (Spencer et al., 2004; Gabbett and Mulvey, 2008; Caprino et al., 2012; Makaje et al., 2012; Reardon et al., 2017). Another example is the concept of repeated high-intensity effort, which has been defined as three or more sprint or collision exertions during the same passage of gameplay with fewer than 21 s between each exertion (Reardon et al., 2017). Contextualized in team sports, repeated-sprint ability is defined as the ability to maintain sprint speed in the course of a game (Spencer et al., 2004), and while it is commonly accepted as a key component in high performance in futsal (Makaje et al., 2012), basketball (Caprino et al., 2012), field hockey (Spencer et al., 2004), and soccer (Gabbett and Mulvey, 2008), Gabbett (2012) reported that in collision sports such as rugby league, players were only exposed to an average of 1 repeated-sprint bout per match compared to an average of nine repeated high-intensity-effort bouts per player per match.

Although some authors have highlighted the importance of monitoring peak external workload intensities (Fox et al., 2020) in seeking to identify how many times in and between matches players perform a volume of activity that is not overly dissimilar to the identified peak period of activity (Carling et al., 2019), no research has attempted to shed some light on whether these peak demands are “one-off” (Carling et al., 2019) or present repeatedly in the course of a single match. Substantial match-to-match variability in the peak demands of competition (coefficient of variation = ∼24%) has been reported (Carling et al., 2016), suggesting that a single match may entail more than one exposure to a scenario that is very close to the most demanding scenario, hereinafter referred to as high-demanding scenario and very high-demanding scenario. This therefore renders it even more necessary to explore the possible repetition of these scenarios during a match and the training process thoroughly.

Understanding the frequency with which a player is required to manage periods with high and very high external loads that are very close to the most demanding scenario could be equally as important as the match’s most demanding scenario concept. This new concept could guide strength and conditioning coaches to a more solid and systematic methodology when planning and structuring training loads during the weekly training program, particularly in a structured microcycle, understood as the training unit imparted between competitions, where also competition loads are the most important factor conditioning the rest of the sessions into systematic phases within the microcycle (Tarragó et al., 2019). An accurate periodization of these training loads is a key factor in achieving the necessary psychological and physiological adaptations that will improve individual and team performance (Akenhead et al., 2016), while reducing the likelihood of illness, injury, and non-functional overreaching (Bourdon et al., 2017).

Therefore, the main objectives of this study were (I) to describe the repetition of high- and very high-demanding scenarios of match play of an elite futsal team of the Spanish 1st Division Liga Nacional de Fútbol Sala and (II) to check if these high- and very high-demanding scenarios were represented within one competition match weekly microcycles and how they were distributed among the different training sessions.

## Materials and Methods

### Design

A retrospective observational study was carried out during the 2018–2019 competitive season, and external load data were collected through an UWB electronic performance tracking system (WIMU PRO^TM^, Realtrack Systems, Almeria, Spain). The data were collected from 13 official matches and 6 in-season training weeks with only one match per week and 4 days of training session prior to the match. The data from goalkeepers and players who played less than 5 min in the matches or did not complete the training session or match due to an injury were excluded, as were data stemming from a pre- or post-session court test. The games and training sessions analyzed were always played on the same court (always home) in the same environmental conditions. Table 1 presents the duration of each training session and match over a 1-week period and the distribution of a total of 347 files analyzed.

**TABLE 1:** The duration and the total number of files across different sessions.

**Match day**	**Duration (hours:minutes)**	**Total files (number of games and participants)**
MD-4	01:41 ± 00:12	59
MD-3	01:39 ± 00:09	55
MD-2	01:37 ± 00:04	57
MD-1	01:25 ± 00:06	60
MD	01:33 ± 00:06	116

**MD-X, match day minus days before game*.*

### Subjects

Thirteen professional elite futsal players (age: 28.8 ± 2.4 years, weight: 73.7 ± 6.2 kg, height: 175.9 ± 5.9 cm, all measurements mean ± standard deviation) from a team that competes in the premier Spanish futsal league Liga Nacional de Fútbol Sala and in the UEFA Futsal Champions League participated voluntarily in the study. The data analyzed stemmed from daily player monitoring in which player activities are routinely measured in the course of the season. The experimental procedures used in this study were in accordance with the Declaration of Helsinki and were approved by the local Ethics and Scientific Committee. All the players provided their informed consent before participating.

### Procedures

The number of competition matches per week, days between matches, and the physical status of the players and the team, among other conditioning factors, affected the structure of every weekly microcycle throughout the season, requiring that the technical staff adjust contents according to the Club’s Structured Training Methodology (Tarragó et al., 2019) adapted to indoor team sports. Due to all these variations, this study only drew data from the weeks when players had only one match at the weekend and four consecutive training sessions (with a clear focus on the match) before the match. External training load data were analyzed with regard to the number of days prior to the match [match day (MD) minus X days] (Akenhead et al., 2016). The training sessions of the microcyles analyzed in this research were always comprised of an integrated content (i.e., tactical, technical, and physical factors were amalgamated) and are described below: MD-4 was the first training in the microcycle. The main goals of this session were to develop player strength and power capabilities through an initial individualized functional strength program performed at the gym followed by small-sided games with goalkeepers on the court. The MD-3 training session focused on developing the team’s playing model. The session was structured in two main blocks, firstly addressing the transitional moments of the game through numerical advantage and disadvantage situations and secondly by working on the defensive system with repeat 4v4 situations conditioned by coach-specified constraints. The main purpose of MD-2 was to tactically and physically prepare the team and the players for the following competition match. This session consisted of working on the team’s game model through real match situations by playing short matches (between 3 and 4 bouts depending on the length of each one) with different preestablished conditioning factors (i.e., point in the game, score, number of faults of each team, etc.). Each short match sought to reproduce similar playing rotations (i.e., point and length of playing time between resting periods for a player in a match) as they usually occur during the match. MD-1 was the day before the competition and was the shortest of all the sessions. It was characterized by using 4v4 (with goalkeepers) games in reduced spaces, emphasizing repeat set pieces (corner kicks and sidekicks), finishing the session with 5v4 tactical situations. The main goals of these sessions remained consistent across the competitive season for all microcycles with only one match per week and 4 days of training session prior to the match.

Data logging was carried out with a local positioning system (WIMU PRO^TM^, Realtrack Systems SL) and its corresponding software (SPRO^TM^, Realtrack Systems SL, version 946). The devices were placed in the upper part of the back, in tight-fitting harnesses. The WIMU PRO^TM^ is equipped with four 3D accelerometers (full-scale out output ranges are ±16, ±16, ±32, and ±400 g. 100 Hz sample frequency), three gyroscopes (8,000°/s full-scale out output range. 100 Hz sample frequency), a 3D magnetometer (100 Hz sample frequency), a GPS (10 Hz sample frequency), and a UWB (18 Hz sample frequency). The UWB system was installed on the court as follows: 6 antennae with UWB technology were fixed 5 m from the court perimeter line. The WIMU PRO system presented better accuracy (bias: 0.57–5.85%), test–retest reliability [% technical error of measurement (% TEM): 1.19] and inter-unit reliability (bias: 0.18) in determining distance than GPS technology (bias: 0.69–6.05%; % TEM: 1.47; bias: 0.25) (Bastida Castillo et al., 2018). More recently, the WIMU PRO system presented a high intra-class correlation coefficient (ICC) value for the x-coordinate (0.65), a very high one for the *y*-coordinate (0.85), and a good % TEM: 2 (Bastida Castillo et al., 2019).

The variables selected to describe the external load scenarios were classified in locomotor distance variables [total distance covered (m⋅min^–1^) and distance covered above 0.02 instantaneous Player Load (vector magnitude expressed as the square root of the sum of the squared instantaneous rates of change in acceleration in each of the 3 planes divided by 100) in arbitrary units (>0.02 AU; m⋅min^–1^)], locomotor velocity variables [absolute high-speed running (distance covered above 18 km⋅h^–1^; m⋅min^–1^), and relative high-speed running (distance covered above 85% of individual maximum speed recorded in training sessions or matches; m⋅min^–1^)]) and neuromuscular variables [number of high-intensity accelerations (>2 m⋅s^–2^; n⋅min^–1^) and number of high-intensity decelerations (>2 m⋅s^–2^; n⋅min^–1^)] following the Zurutuza and Castellano (2020) external load classification.

The raw data from the EPTS and its software SPRO^TM^ were computed with RStudio version 1.2.5033 (RStudio, Inc.). The data computation procedures were structured in five steps, which were always applied for each player and each variable. The first step was to determine the most demanding scenario of each match. The rolling average method with a 1-min time window was used, and the results made it possible to establish a data frame of individual maximum values. The second step was to determine a reference value (100% most demanding scenario of the MD value). For this purpose, a mean of the top three observations was taken in order to smooth possible outliers. The third step was to establish thresholds to count the number of high-demanding scenarios, very high-demanding scenarios, and high plus very high-demanding scenarios. A lower limit threshold of 90% of the reference value of the most demanding scenario was established for very high-demanding scenarios, and a lower and upper limit threshold of 80–90% of the reference value of the most demanding scenario was established for high-demanding scenarios. The fifth and last step was to re-conduct a rolling average and save not the most demanding scenario but rather all the scenarios within the defined thresholds. The final output was the total number of high-, very high-, and high plus very high-demanding scenarios for each training session and match, for each player and variable.

### Statistical Analysis

All statistical analyses were conducted with RStudio version 1.2.5033 (RStudio, Inc.). Descriptive data were reported as mean ± standard deviation. The data failed all the tests for homogeneity of variance (Levene’s test) and for normality (Shapiro–Wilk’s test). To perform the hypothesis test to assess the differences between MDs, a bootstrap CI approach was used because the assumptions of this method were aligned with our data (Wilcox, 2010). A residual resampling model, with 10,000 bootstrap samples and 95% bias-corrected and accelerated method (BCa 95% CI), was used to calculate the CI of *F*-values of repeated-measure ANOVA for each variable and established that the null hypothesis, that there were no differences, was true if 1 fell within the CI limits (Plonsky, 2015). The same bootstrap CI approach with a simple resampling model was used to evaluate the *post hoc* pairwise comparisons in the variables with differences between MDs. The mean difference in the number of scenarios was computed and presented as percentage change rate (Δ%Scenario); data were presented as mean difference and BCa 95% CI in contrast plots (Ho et al., 2019; Jovanovic, 2020). All the reported *P*-values were the likelihoods of observing the absolute effect sizes if the null hypothesis of zero difference was true (Plonsky, 2015).

## Results

The descriptive data of the number of high- and very high-demanding scenarios are represented in Table 2. The MD-2 training session and the MD were the 2 days in the microcycle with the greatest number of scenarios. Table 3 presents the results of the bootstrap ANOVA. In the very high-demanding scenario threshold, only in high-intensity decelerations were no significant differences found; and in the high demanding scenario threshold, no significant differences were found in absolute and relative high-speed running.

**TABLE 2:** Number (mean ± standard deviation) of scenarios for each type, match day, and variable analyzed.

**Scenario type**	**Match day**	**Absolute high-speed running**	**Relative high-speed running**	**Total distance**	**Player load**	**High-intensity accelerations**	**High-intensity decelerations**
High + very high-demanding scenarios	MD-4	0.53 ± 0.80	0.49 ± 0.95	2.86 ± 2.05	0.98 ± 1.28	1.34 ± 2.43	1.03 ± 1.92
	MD-3	0.47 ± 0.69	0.35 ± 0.62	2.96 ± 2.93	0.84 ± 1.08	0.93 ± 1.51	0.65 ± 1.38
	MD-2	1.02 ± 1.04	1.00 ± 0.91	4.09 ± 2.47	1.65 ± 1.54	0.54 ± 1.04	0.51 ± 1.15
	MD-1	0.18 ± 0.54	0.17 ± 0.46	0.43 ± 0.74	0.13 ± 0.34	0.22 ± 0.64	0.13 ± 0.39
	MD	0.63 ± 0.91	0.47 ± 0.74	4.75 ± 2.45	2.30 ± 1.79	1.41 ± 1.64	1.12 ± 1.26
Very high-demanding scenarios	MD-4	0.36 ± 0.66	0.31 ± 0.73	0.58 ± 0.88	0.25 ± 0.68	0.71 ± 1.73	0.53 ± 1.24
	MD-3	0.24 ± 0.51	0.22 ± 0.50	0.56 ± 0.76	0.25 ± 0.48	0.38 ± 0.76	0.38 ± 0.95
	MD-2	0.68 ± 0.89	0.68 ± 0.85	1.00 ± 1.34	0.75 ± 1.15	0.18 ± 0.57	0.25 ± 0.85
	MD-1	0.08 ± 0.28	0.10 ± 0.35	0.10 ± 0.35	0.05 ± 0.22	0.07 ± 0.31	0.10 ± 0.35
	MD	0.37 ± 0.68	0.32 ± 0.58	1.38 ± 1.51	0.77 ± 0.98	0.58 ± 0.91	0.51 ± 0.95
High-demanding scenarios	MD-4	0.17 ± 0.42	0.19 ± 0.39	2.29 ± 1.58	0.73 ± 0.91	0.63 ± 1.31	0.51 ± 0.97
	MD-3	0.24 ± 0.51	0.13 ± 0.39	2.40 ± 2.48	0.58 ± 0.88	0.55 ± 1.03	0.27 ± 0.89
	MD-2	0.33 ± 0.58	0.32 ± 0.51	3.09 ± 1.86	0.89 ± 0.94	0.37 ± 0.64	0.26 ± 0.67
	MD-1	0.10 ± 0.35	0.07 ± 0.25	0.33 ± 0.63	0.08 ± 0.28	0.15 ± 0.40	0.03 ± 0.18
	MD	0.26 ± 0.53	0.15 ± 0.42	3.37 ± 1.88	1.53 ± 1.29	0.83 ± 1.23	0.61 ± 0.83

**MD-X, session minus days before the game.**

**TABLE 3:** Bootstrap ANOVA results for each scenario threshold.

**Variable**	***F***	Bootstrap*			
		**Bias**	**Std. error**	**BCa 95% CI lower**	**BCa 95% CI upper**
**ANOVA for high + very high-demanding scenarios**					
Absolute high-speed running	8.186	3.016	3.482	2.341	11.661
Relative high-speed running	11.859	3.940	4.268	3.375	15.766
Total distance	52.897	14.099	10.678	33.833	57.891
Player load	36.326	9.958	8.690	19.535	42.101
High-intensity accelerations	11.828	4.152	4.681	2.665	16.236
High-intensity decelerations	8.027	3.183	3.676	1.834	11.507
**ANOVA for very high-demanding scenarios**					
Absolute high-speed running	7.144	2.819	3.366	1.660	10.586
Relative high-speed running	8.250	3.062	3.632	1.524	12.077
Total distance	17.800	5.478	5.626	8.740	22.561
Player load	15.448	4.964	5.242	5.511	20.086
High-intensity accelerations	6.350	2.932	3.587	1.066	9.808
High-intensity decelerations	3.198	1.857	2.245	0.186	5.564
**ANOVA for high-demanding scenarios**					
Absolute high-speed running	1.908	1.513	1.750	0.024	3.626
Relative high-speed running	3.345	1.860	2.331	0.247	5.784
Total distance	38.673	10.611	9.085	21.415	44.503
Player load	25.067	7.159	6.667	11.069	29.967
High-intensity accelerations	8.795	3.412	3.998	1.639	12.807
High-intensity decelerations	8.195	3.085	3.645	1.638	11.776

The null hypothesis was rejected if 1 did not fall within BCa 95% CI limits. * Bootstrap results are based on 10,000 bootstrap samples.

**FIGURE 1:**
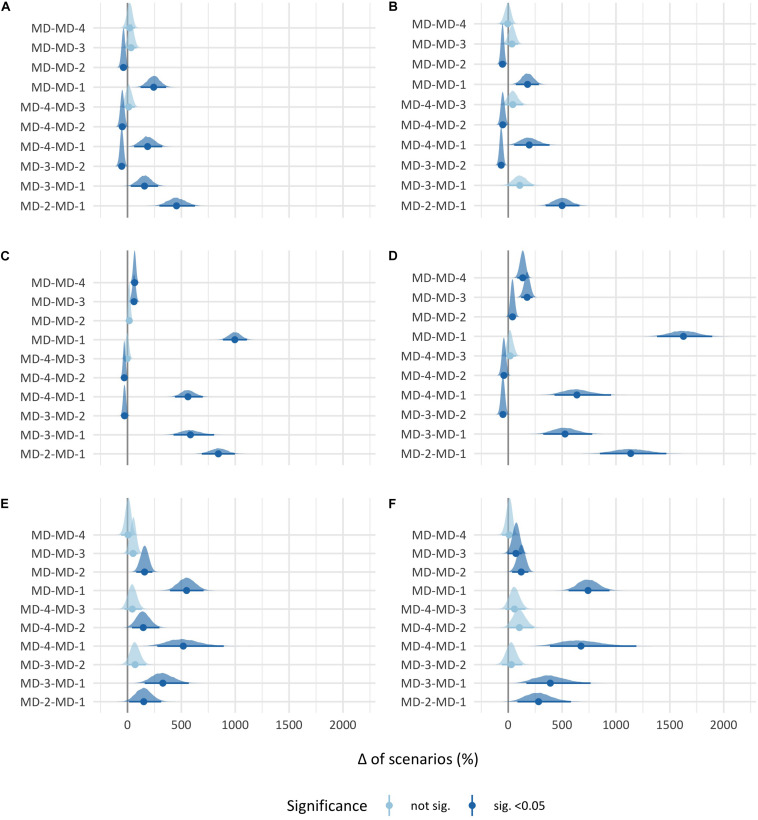
95% Confidence interval percentage change of high plus very high-demanding scenarios between match days. Each point represents the mean percentage change, and each black line through the point represents the 95% confidence interval of the percentage change between the two match days. **(A)** Absolute high-speed running. **(B)** Relative high-speed running. **(C)** Total distance. **(D)** Player load. **(E)** High-intensity accelerations, **(F)** high-intensity decelerations.

The *post hoc* analysis of the high plus very high-demanding scenario threshold is displayed in Figure 1. Briefly, the results showed that in absolute and relative high-speed running, MD-2 had a significantly greater number of scenarios than the other days (the greatest difference was found between MD-2 and MD-1, with a mean difference of 0.84 [BCa 95% CI: 0.54–1.15] in absolute high-speed running and 0.83 [BCa 95% CI: 0.58–1.10] in relative high-speed running), and that MD-1 had significantly fewer scenarios than the other days except in relative high-speed running between MD-3 and MD-1 (the greatest difference was found between MD and MD-1, with a mean difference of 0.45 [BCa 95% CI: 0.22–0.66] in absolute high-speed running and 0.29 [BCa 95% CI: 0.12–0.47] in relative high-speed running). Except between MD and MD-2 in total distance, MD had a significantly greater number of total distance and player load scenarios than the other days (the greatest difference was found between MD and MD-1, with a mean difference of 4.31 [BCa 95% CI: 3.84–4.80] in total distance and 2.17 [BCa 95% CI: 1.84–2.52] in player load) and MD-2 had a significantly greater number of scenarios than the other days (except with MD) (the greatest difference was found between MD-2 and MD-1, with a mean difference of 3.65 [BCa 95% CI: 2.99–4.32] in total distance and 1.52 [BCa 95% CI: 1.13–1.95] in player load), and MD-1 had significantly fewer scenarios than all other days (the greatest difference was found between MD-3 and MD-1, with a mean difference of 2.53 [BCa 95% CI: 1.85–3.48] in total distance and 0.70 [BCa 95% CI: 0.43–1.04] in player load). Finally, in high-intensity accelerations and decelerations, MD-1 had significantly fewer scenarios than all other days (the greatest difference was found between MD and MD-1, with a mean difference of 1.19 [BCa 95% CI: 0.86–1.53] in high-intensity accelerations and 0.99 [BCa 95% CI: 0.75–1.25] in high-intensity decelerations).

**FIGURE 2:**
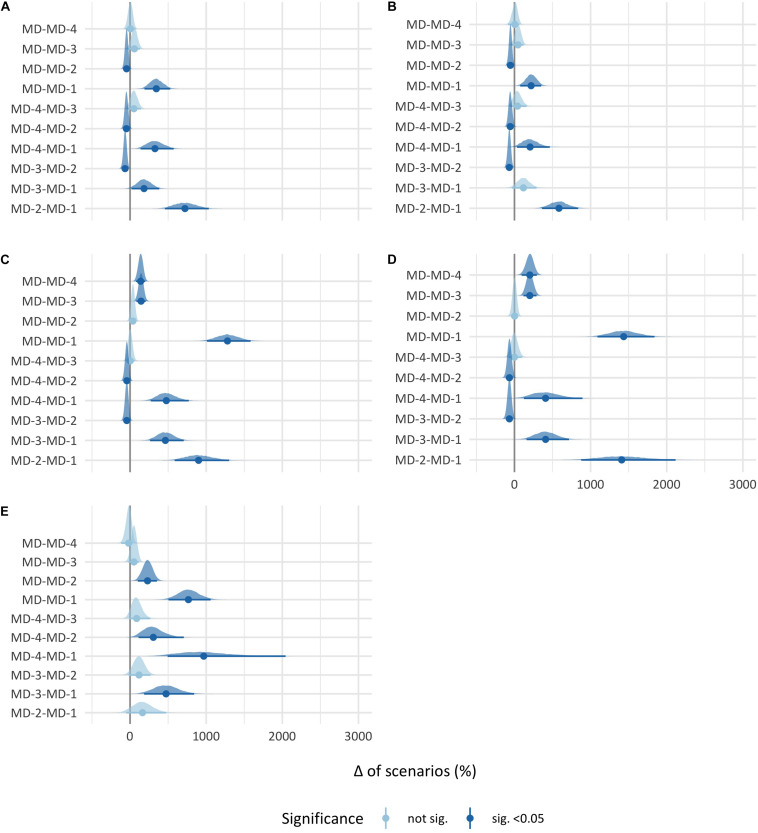
95% Confidence interval percentage change of very high-demanding scenarios between match days. Each point represents the mean percentage change, and each black line through the point represents the 95% confidence interval of the percentage change between the two match days. **(A)** Absolute high-speed running. **(B)** Relative high-speed running. **(C)** Total distance. **(D)** Player load. **(E)** High-intensity accelerations.

The *post hoc* analysis of the very high-demanding scenario threshold is presented in Figure 2. Briefly, the pairwise comparisons presented the same direction differences as those found in the high plus very high-demanding scenario threshold in absolute and relative high-speed running, albeit to a lesser extent. In total distance and player load, except the comparisons between MD and MD-2, the comparisons between the days were significantly different in the same direction as in the high plus very high-demanding scenario threshold.

**FIGURE 3:**
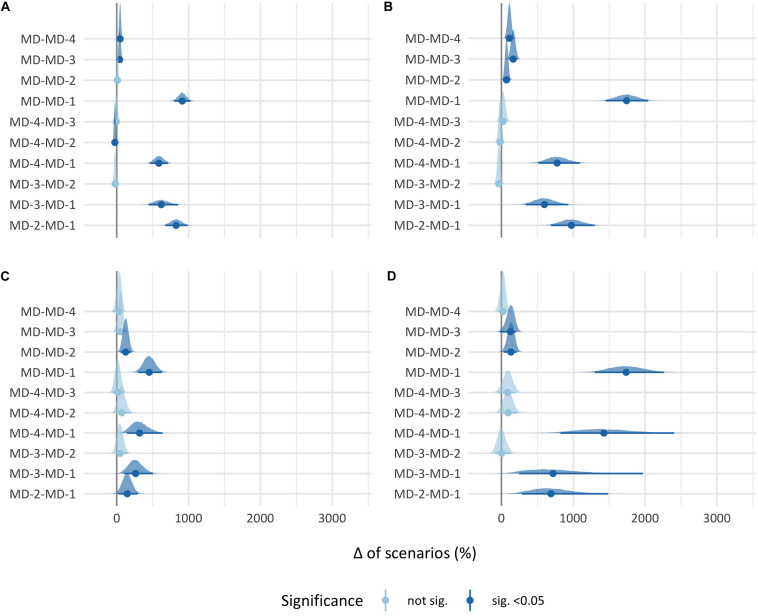
95% Confidence interval difference of number of high-demanding scenarios between match days. Each point represents the mean difference, and each black line through the point represents the 95% confidence interval of the difference between the two match days. **(A)** Total distance. **(B)** Player load. **(C)** High-intensity accelerations. **(D)** High-intensity decelerations.

Finally, the *post hoc* analysis of the high-demanding scenario threshold is presented in Figure 3. Briefly, in total distance and player load, the differences between MD and all the training days were in the same direction of the high plus very high-demanding scenario threshold; MD-2 only had significant differences with MD-4 and MD-1 in total distance and with MD-1 in player load; and MD-1, as in the high plus very high-demanding scenario threshold, had significant differences with the other days. In high-intensity decelerations, the direction and significance of the differences were the same as in the high plus very high demanding scenario threshold but of less magnitude.

## Discussion

This is the first study to quantify the repetition of high and very high-demanding scenarios of match play in a professional elite futsal team. It is also the first to investigate whether these scenarios are represented throughout typical one-match weekly microcycles and how they are distributed across the different training sessions. The main findings were as follows: (a) futsal players were exposed to more than one high and very high-demanding scenario in a single match, with locomotor distance variables presenting higher repeatability than neuromuscular and locomotor velocity variables, and (b) when these scenarios are compared to training, the number of exposures for players was variable-dependent and training session-dependent.

### Quantification of High- and Very High-Demanding Scenarios

One of the major findings of this study was that in a single futsal match, the repetition of high- and very high-demanding scenarios of total distance (3.37 ± 1.88 and 1.38 ± 1.51, respectively) was higher than the other variables analyzed. Additionally, repeat highly demanding bouts of player load were observed in this study (1.53 ± 1.29 high-demanding scenarios and 0.77 ± 0.98 very high-demanding scenarios), suggesting that peak locomotor distance demands are highly repetition-prone during futsal matches. It remains unclear to which degree these results may be attributed to the intrinsic low-intensity nature of the locomotor distance variables, to the idiosyncrasy of the short stoppages and interruptions during futsal matches (i.e., outsides, corner-kicks, etc.) in which players continue to move even when the ball is not in play, or to the playing model of the team, which is characterized by a fast-paced game play.

It is now generally accepted that the small dimensions of indoor sports courts (a futsal court is 40 m long and 20 m wide) are a limiting factor when referring to locomotor velocity variables. Small relative areas (80 square meters per player in futsal matches) are known to under-stimulate the distance covered at high speed (Martín-García et al., 2020). This is consistent with our findings and may therefore explain why absolute and relative high-speed running exhibited the lowest number of repetitions of high- and very high-demanding scenarios during futsal matches (0.63 ± 0.91 high plus very high-demanding scenarios of absolute high-speed running and 0.47 ± 0.74 high plus very high-demanding scenarios of relative high-speed running).

This study also revealed that the team analyzed was more susceptible to the repetition of high- and very high-demanding scenarios of neuromuscular variables such as high-intensity accelerations and decelerations in a match (0.83 ± 1.23 and 0.58 ± 0.91 high-intensity accelerations and 0.61 ± 0.83 and 0.51 ± 0.95 high-intensity decelerations in the high- and very high-demanding scenario thresholds, respectively) than the repetition shown in relation to locomotor velocity variables. These results indicate that the overall repeatability of high- and very high-demanding scenarios in a futsal match for the team studied is lower when high-intensity variables are analyzed. However, we acknowledge, in line with the ideas of Martín-García et al. (2018a), that other activities occur within the most demanding scenario of a match. For example, as reported by Martín-García et al. (2018a), during the most demanding scenario of total distance for a 1-min time window, professional soccer players covered an average of 191.6 ± 19.7 m⋅min^–1^, together with 38.3 ± 23.1 m⋅min^–1^ of distance above 19.8 km⋅h^–1^, 2.6 ± 1.6 high-intensity accelerations ⋅min^–1^ (>3 m⋅s^2^), and 3.5 ± 1.6 high-intensity decelerations min^–1^ (<−3 m⋅s^2^); hence, future research should further investigate other load variable behaviors while a player is exposed to high- and very high-demanding scenarios of any selected variable.

### High- and Very High-Demanding Scenarios in the Structured Microcycle

When the repetition of high- and very high-demanding scenarios versus the number of training days prior to a match was analyzed, this study observed that MD-2 was the training session that best represented match high- and very high-demanding scenario repetition in locomotor distance variables, with players performing 4.09 ± 2.47 repetitions of total distance high plus very high-demanding scenarios, matching those found in the match or even surpassing the match in locomotor velocity variables (difference between MD-2 and MD of 0.39 [BCa 95% CI: 0.08–0.72] in absolute high-speed running and 0.53 [BCa 95% CI: 0.28–0.81] in relative high-speed running, in both high- and very high-demanding scenario threshold). We speculate that this parallelism between MD-2 and the match may be attributable to the nature of this type of session, in which coaches implement simulated competition sequences according to the team’s game model. In line with this idea, the MD-4 particularities of the team analyzed, involving small-sided games intended to develop strength and power, seem to provide a rationale for it being the training session presenting the highest number of repeated high- and very high-demanding scenarios of high-intensity accelerations and decelerations, while also matching the demands of competition in terms of highly demanding peaks of neuromuscular activity. In this study, repeat exposure to these bouts gradually decreased on the days leading up to the match. This tallies with the findings of Martín-García et al. (2018b), who reported a progressive decrease in all metrics when quantifying the external load of a soccer team using the same structured microcycle as the one analyzed in this investigation. The small-sided games commonly used for the team studied during MD-4 would also account for the significantly lower repetition of high- and very high-demanding scenarios of absolute and relative high-speed running when compared to the match and to MD-2. Curiously, MD-3 exhibited a strong similarity with MD-4 for all variables studied, regardless of the different objectives and contents proposed by the coaches for both training session types. Another interesting finding of this study was that regardless of the variable analyzed, the number of high- and very high-demanding scenarios in MD-1 diminished compared to the rest of the training sessions. These results seem to agree with those of prior studies quantifying the external load of training microcycles in soccer (Malone et al., 2015; Owen et al., 2017; Martín-García et al., 2018b), which reported that MD-1 had the lowest external load of all training sessions within the microcycle. As suggested by Malone et al. (2015), this drop in MD-1 could be considered as a tapering-off strategy by coaches geared toward increasing player readiness for the forthcoming match.

Although this study is based on a single futsal team, the findings provide a new understanding as to how many times players have to cope with periods of activity that are close to the most demanding scenario of match play during futsal matches. One limitation of the study is the small number of training weeks included, since only six training weeks matched the inclusion criteria due to the team’s busy calendar. Additionally, and given that an UWB positioning system was required to record the match data, only 13 official matches from a single team were included in the study. In spite of its limitations, the results of this study lay the foundations for future research including internal load variables together with the description of the activities performed by the players during the high and very high demanding scenarios in a given tactical context.

## Conclusion

The analysis of the repetition of high- and very high-demanding scenarios undertaken in this study support the idea that high demands of competition present repeatedly in the course of a single match instead of being a “one off” event (Carling et al., 2019), providing a new method to monitor and quantify the most demanding scenario demands in match play. From the training prescription standpoint, the findings of this study show that the team in question experience the overall repeatability of high- and very high-demanding scenarios performed during competition throughout each systematic phase of the structured microcycle by stressing the high- and very high-demanding scenarios of neuromuscular and locomotor velocity variables at the beginning of the week and the high- and very high-demanding scenarios of locomotor distance variables 2 days prior to the match, leaving the closest training session to the match without stressing any of these scenarios.

## Data Availability Statement

The raw data supporting the conclusions of this article will be made available by the authors, without undue reservation, to any qualified researcher.

## Ethics Statement

The studies involving human participants were reviewed and approved by Ethics Committee for Clinical Research of the Catalan Council, Generalitat de Catalunya, Spain. The patients/participants provided their written informed consent to participate in this study.

## Author Contributions

JI participated in the design of the study, contributed to data collection and interpretation of results, and contributed to the manuscript writing. DF participated in the design of the study, contributed to data reduction, analysis, and interpretation of results, and contributed to the manuscript writing. XR participated in the design of the study, contributed to data reduction, analysis, and interpretation of results. GC contributed to the manuscript writing. JT participated in the design of the study and contributed to the manuscript writing. All authors have read and approved the final version of the manuscript and agree with the order of presentation of the authors.

## Conflict of Interest

The authors declare that the research was conducted in the absence of any commercial or financial relationships that could be construed as a potential conflict of interest.

## Abbreviations

UWB: ultra-wideband
EPTS: electronic performance and tracking system
UEFA: Union of European Football Associations
MD: match day
MD-x: match day [MD] minus X days
GPS: global positioning system
CI: confidence intervals.

## References

[B1] Akenhead R. Harley J. A. Tweddle S. P. (2016). Examining the External Training Load of an English Premier League Football Team With Special Reference to Acceleration. *J. Streng. Condit. Res.* 30 2424–2432. 10.1519/JSC.0000000000001343 26817740

[B2] Bastida Castillo A. Gómez Carmona C. De la Cruz Sánchez E. Reche Royo X. Ibáñez S. Pino Ortega J. (2019). Accuracy and Inter-Unit Reliability of Ultra-Wide-Band Tracking System in Indoor Exercise. *Appl. Sci.* 9:939 10.3390/app9050939

[B3] Bourdon P. C. Cardinale M. Murray A. Gastin P. Kellmann M. Varley M. C. (2017). Monitoring Athlete Training Loads: Consensus Statement. *Int. J. Spor. Physiol. Perform.* 12 S2161–S2170. 10.1123/IJSPP.2017-0208 28463642

[B4] Caprino D. Clarke N. D. Delextrat A. (2012). The effect of an official match on repeated sprint ability in junior basketball players. *J. Spor. Sci.* 30 1165–1173. 10.1080/02640414.2012.695081 22697579

[B5] Carling C. Bradley P. McCall A. Dupont G. (2016). Match-to-match variability in high-speed running activity in a professional soccer team. *J. Spor. Sci.* 34 2215–2223. 10.1080/02640414.2016.1176228 27144879

[B6] Carling C. McCall A. Harper D. Bradley P. S. (2019). Comment on: “The Use of Microtechnology to Quantify the Peak Match Demands of the Football Codes: A Systematic Review.”. *Spor. Med.* 49 343–345. 10.1007/s40279-018-1032-z 30506338

[B7] Castillo A. Gómez Carmona C. D. De la cruz sánchez E. Pino Ortega J. (2018). Accuracy, intra- and inter-unit reliability, and comparison between GPS and UWB-based position-tracking systems used for time-motion analyses in soccer. *Eur J. Spor. Sci.* 18 450–457. 10.1080/17461391.2018.1427796 29385963

[B8] Delaney J. A. Thornton H. R. Burgess D. J. Dascombe B. J. Duthie G. M. (2017). Duration-specific running intensities of Australian Football match-play. *J Sci. Med. Spor.* 20 689–694. 10.1016/j.jsams.2016.11.009 28131505

[B9] Fernández D. Novelles A. Tarragó R. Reche X. (2020). Comparing the Most Demanding Passages of Official Matches and Training Drills in Elite Roller Hockey. *Apunts. Educa. Física Deport.* 2 77–80. 10.5672/apunts.2014-0983.es.(2020/2).140.11

[B10] Fox J. Conte D. Stanton R. Mclean B. Scanlan A. (2020). The Application of Accelerometer-Derived Moving Averages to Quantify Peak Demands in Basketball: A Comparison of Sample Duration, Playing Role, and Session Type. *J. Streng. Condit. Res.* 2020 1–1. 10.1519/JSC.0000000000003486 34846331

[B11] Gabbett T. J. (2012). Sprinting Patterns of National Rugby League Competition. *J. Streng. Condit. Res.* 26 121–130. 10.1519/JSC.0b013e31821e4c60 22158144

[B12] Gabbett T. J. Jenkins D. G. Abernethy B. (2012). Physical demands of professional rugby league training and competition using microtechnology. *J. Sci. Med. Sport* 15 80–86. 10.1016/j.jsams.2011.07.004 21820959

[B13] Gabbett T. J. Kennelly S. Sheehan J. Hawkins R. Milsom J. King E. (2016). If overuse injury is a ‘training load error’, should undertraining be viewed the same way? *Br. J. Spor. Med.* 50 1017–1018. 10.1136/bjsports-2016-096308 27251895

[B14] Gabbett T. J. Mulvey M. J. (2008). Time-Motion Analysis of Small-Sided Training Games and Competition in Elite Women Soccer Players. *J. Streng. Condit. Res.* 22 543–552. 10.1519/JSC.0b013e3181635597 18550972

[B15] Ho J. Tumkaya T. Aryal S. Choi H. Claridge-Chang A. (2019). Moving beyond P values: Data analysis with estimation graphics. *Nat. Methods* 16 565–566. 10.1038/s41592-019-0470-3 31217592

[B16] Jovanovic M. (2020). *bmbstats: Magnitude-based statistics for sports scientists (1st ed.).* United Kingdom: Self Publishing.

[B17] Makaje N. Ruangthai R. Arkarapanthu A. Yoopat P. (2012). Physiological demands and activity profiles during futsal match play according to competitive level. *J. Spor. Med. Phys. Fit.* 52 366–374. 22828458

[B18] Malone J. J. Di Michele R. Morgans R. Burgess D. Morton J. P. Drust B. (2015). Seasonal training-load quantification in elite English premier league soccer players. *Int. J. Spor. Physiol. Perform.* 10 489–497. 10.1123/ijspp.2014-0352 25393111

[B19] Martín-García A. Casamichana D. Díaz A. G. Cos F. Gabbett T. J. (2018a). Positional Differences in the Most Demanding Passages of Play in Football Competition. *J. Spor. Sci. Med.* 17 563–570. PMC6243617 30479524

[B20] Martín-García A. Gómez Díaz A. Bradley P. S. Morera F. Casamichana D. (2018b). Quantification of a Professional Football Team’s External Load Using a Microcycle Structure. *J. Stren. Condit. Res.* 32 3511–3518. 10.1519/JSC.0000000000002816 30199452

[B21] Martín-García A. Castellano J. Villanueva A. M. Gómez-Díaz A. Cos F. Casamichana D. (2020). Physical Demands of Ball Possession Games in Relation to the Most Demanding Passages of a Competitive Match. *J. Sport. Sci. Med.* 19 1–9. PMC7039032 32132822

[B22] Owen A. L. Djaoui L. Newton M. Malone S. Mendes B. (2017). A contemporary multi-modal mechanical approach to training monitoring in elite professional soccer. *Sci. Med. Football* 1 216–221. 10.1080/24733938.2021.1942539 35475739

[B23] Plonsky L. (2015). *Advancing Quantitative Methods in Second Language Research (1st ed.).* United Kingdom: Routledge.

[B24] Reardon C. Tobin D. P. Tierney P. Delahunt E. (2017). The worst case scenario: Locomotor and collision demands of the longest periods of gameplay in professional rugby union. *PLoS One* 12:e0177072. 10.1371/journal.pone.0177072 28510582 PMC5433699

[B25] Spencer M. Lawrence S. Rechichi C. Bishop D. Dawson B. Goodman C. (2004). Time-motion analysis of elite field hockey, with special reference to repeated-sprint activity. *J. Sport. Sci.* 22 843–850. 10.1080/02640410410001716715 15513278

[B26] Tarragó J. Massafret-Marimón M. Seirul⋅lo F. Cos F. (2019). Training in Team Sports: Structured Training in the FCB. *Apun. Educa. Física Deport.* 3 103–114. 10.5672/apunts.2014-0983.es.(2019/3).137.08

[B27] Vázquez-Guerrero J. Ayala Rodriguez F. Garcia F. Sampaio J. E. (2020). The most demanding scenarios of play in basketball competition from elite Under-18 teams. *Front. Psychol* 11:552. 10.3389/fpsyg.2020.00552 32373001 PMC7187750

[B28] Wilcox R. R. (2010). *Fundamentals of Modern Statistical Methods: Substantially Improving Power and Accuracy Version 2*, 2nd Edn Germany: Springer-Verlag.

[B29] Zurutuza U. Castellano J. (2020). Comparación de la respuesta física, en términos absolutos y relativos a la competición, de diferentes demarcaciones en tareas jugadas de fútbol. *Cuader. De Psicol. Del Deport*. 20(1), 190–200.

